# Chronic Pelvic Pain Due to Residual Ectopic Tissue After Treatment With Methotrexate

**DOI:** 10.7759/cureus.83252

**Published:** 2025-04-30

**Authors:** Allix M Hillebrand, Justin To

**Affiliations:** 1 Minimally Invasive Gynecologic Surgery, Maimonides Medical Center, Brooklyn, USA

**Keywords:** chronic ectopic pregnancy, chronic pelvic pain, ectopic pregnancy, methotrexate, methotrexate therapy for ectopic pregnancy

## Abstract

Ectopic pregnancy is a medical emergency that requires prompt treatment. Treatment options include either medical management with methotrexate or surgical management. There are several risks associated with methotrexate therapy. However, chronic pelvic pain is typically not a risk cited. The case presented is a 27-year-old Hispanic woman, gravida two, para zero with chronic pelvic pain and a history of left tubal ectopic pregnancy successfully treated with methotrexate. Pelvic ultrasound demonstrated a hemorrhagic structure adjacent to the left ovary suspicious for residual ectopic pregnancy tissue. After a period of conservative management, the patient elected for surgical management via laparoscopic left salpingectomy. Pathologic analysis of the specimen confirmed a hyalinized nodule with chronic inflammation, suggestive of remote treatment of ectopic pregnancy. Post-operatively, the patient’s chronic pelvic pain completely resolved. Further research needs to be done on the incidence of chronic pelvic pain following methotrexate treatment for ectopic pregnancy.

## Introduction

Ectopic pregnancy, where a fertilized egg implants outside the uterus, is a medical emergency that requires prompt treatment. Treatment options include either medical or surgical management [1]. Medical treatment typically involves the use of methotrexate, a folate antagonist that stops the growth of the pregnancy tissue. Methotrexate works by inhibiting the rapid cell division of the trophoblast cells. Serum hCG levels are followed to ensure a decline and confirm successful treatment of the ectopic pregnancy [2].

One of the most common risks of methotrexate treatment is incomplete resolution of the pregnancy, leading to persistently elevated hCG levels requiring additional doses of methotrexate or surgical intervention. One of the most significant complications is tubal rupture. Although rare, it can occur if the ectopic pregnancy is not fully resolved by methotrexate, causing life-threatening internal bleeding [3].

Common side effects of methotrexate treatment include gastrointestinal symptoms such as nausea, vomiting, stomatitis, and diarrhea. Acute abdominal pain is also a common symptom post-treatment [4]. However, a literature review yielded a paucity of data regarding chronic pelvic pain after methotrexate treatment. The case presented demonstrates the workup of chronic pelvic pain in a patient treated with methotrexate for ectopic pregnancy.

## Case presentation

The patient is a 27-year-old Hispanic woman, gravida two, para zero with a history of left tubal ectopic pregnancy treated at an outside institution. She had no medical or surgical history. Her gynecologic history was significant for recent antibiotic treatment of an unknown sexually transmitted infection (STI) at an outside institution. Her obstetric history was significant for the antecedent ectopic pregnancy as well as a prior early spontaneous abortion.

The patient was successfully treated with a single dose of methotrexate as evident from a more than 15% drop in serum beta hCG from day four (2534 mIU/mL) to day seven (1911 mIU/mL) as per traditional protocol. Serum beta hCG was followed until it was negative (<0.5 mIU/mL). The patient presented three months after initial treatment to our institution’s outpatient clinic reporting left-sided pelvic pain and dyspareunia. She reported the resumption of normal menses. Vital signs were within normal limits. The urine pregnancy test was negative. Exam was significant for mild left lower quadrant tenderness without rebound and pelvic exam with mild cervical motion tenderness. Differential diagnosis at that time was broad and included gynecologic, urologic, gastrointestinal, and musculoskeletal etiologies. A basic workup, including STI testing, urine culture, and pelvic ultrasound, was completed.

**Figure 1:**
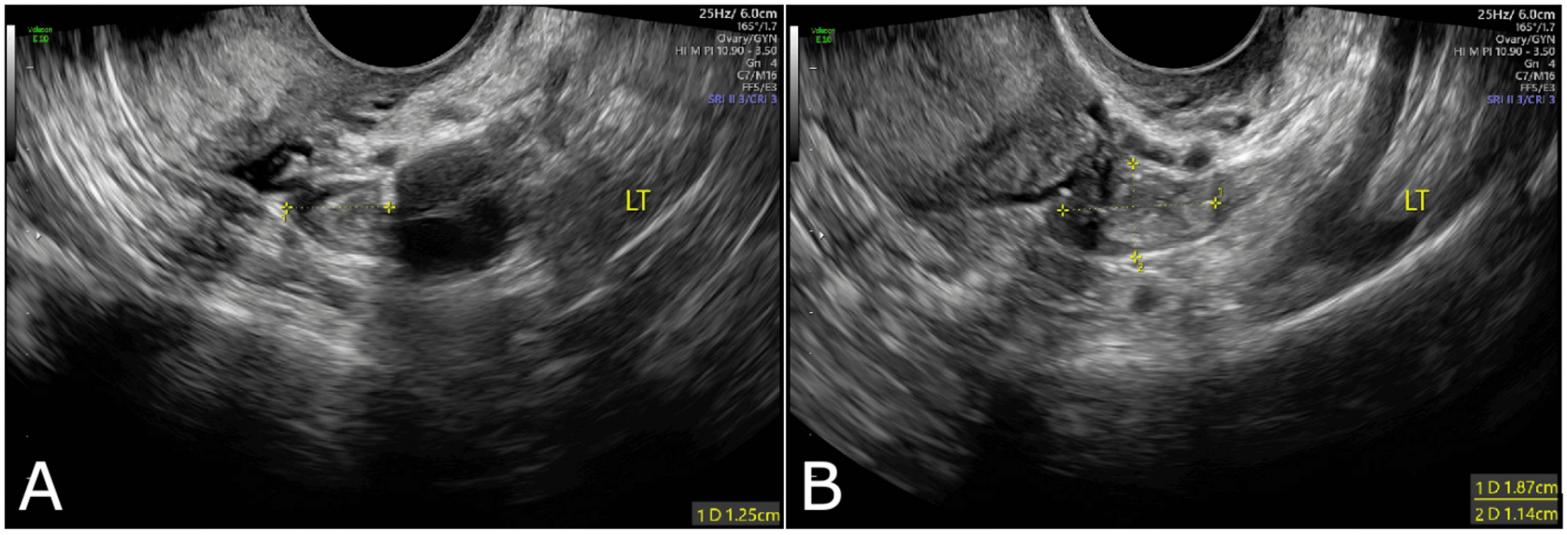
Pelvic Ultrasound Pelvic ultrasound demonstrating a hemorrhagic structure adjacent to the left ovary measuring 1.25 x 1.87 x 1.14 cm.

The patient presented three weeks later for follow-up to review results. She reported no change in her pain. STI and urine testing were negative. Transvaginal pelvic ultrasound showed a 7 cm anteverted uterus and endometrial thickness of 5.8 mm. Bilateral ovaries appeared normal. A hemorrhagic structure adjacent to the left ovary measuring 1.25 x 1.87 x 1.14 cm was suspicious for residual ectopic pregnancy tissue (Figures 1A, 1B).

Differential diagnosis included pain due to residual ectopic pregnancy tissue and less likely pelvic inflammatory disease. She was empirically treated with ceftriaxone, doxycycline, and metronidazole for pelvic inflammatory disease. She was also instructed to take acetaminophen and ibuprofen as needed for pain.

**Figure 2:**
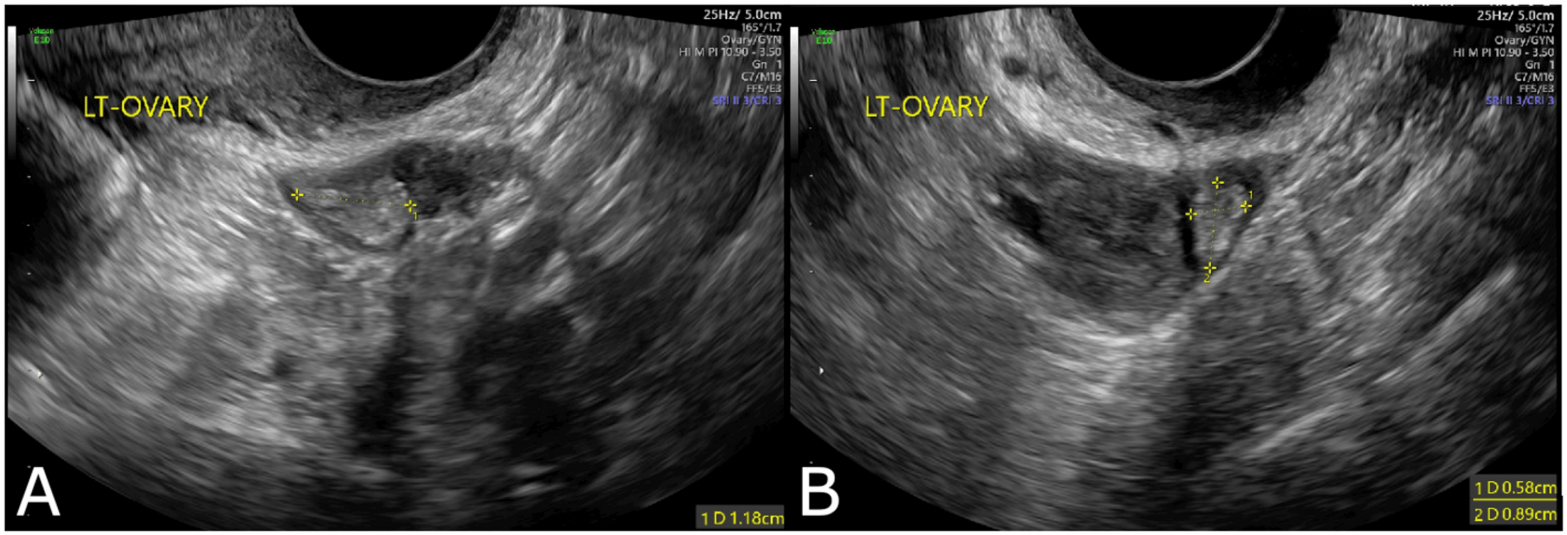
Repeat Pelvic Ultrasound Repeat pelvic ultrasound demonstrating hemorrhagic structure adjacent to the left ovary measuring 1.18 x 0.58 x 0.89 cm.

She returned for re-evaluation two weeks later after completion of antibiotics. The patient reported no change in the pain. She was markedly distressed about how this pain was affecting her life. At this point, the most likely differential diagnosis was pain due to residual ectopic tissue in the left fallopian tube. She was offered expectant management or diagnostic laparoscopy with possible left salpingectomy. Using shared decision making, the patient opted for continued expectant management. If there was no improvement in her pain at her next visit, she would like to pursue surgery. Pelvic ultrasound was repeated and showed an interval decrease in the size of the hemorrhagic structure to 1.18 x 0.58 x 0.89 cm (Figures 2A, 2B).

The patient was again seen for follow-up four weeks later and reported continued left-sided pelvic pain and dyspareunia despite conservative management with acetaminophen and ibuprofen. She elected for surgical management at this time with diagnostic laparoscopy and left salpingectomy.

**Figure 3:**
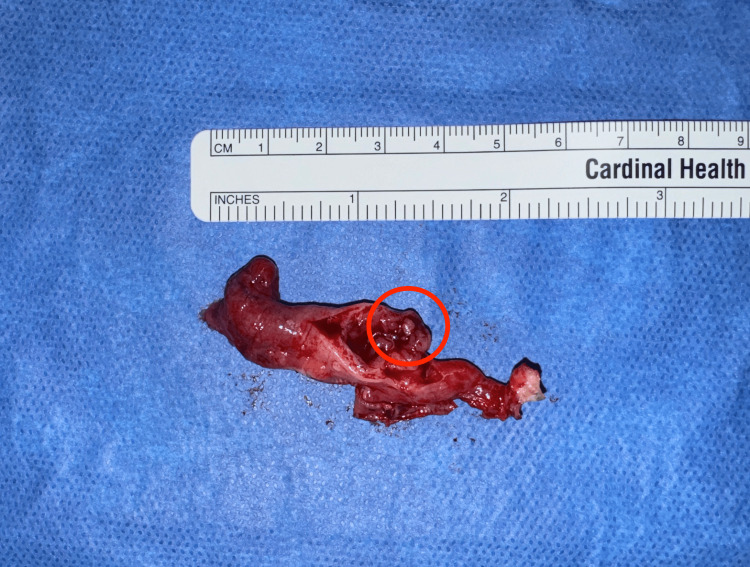
Left Fallopian Tube Gross examination of the left fallopian tube showed a small hard nodule within the ampulla (circled).

The patient presented for surgery approximately seven months after the initial treatment of the ectopic pregnancy. Diagnostic laparoscopy was performed and bilateral adnexa appeared normal. Left salpingectomy was performed by dividing the fallopian tube from the mesosalpinx using bipolar electrocautery. The specimen was removed from the abdomen and examined grossly. There was noted to be a hard nodule in the ampulla of the fallopian tube approximately 0.5 cm in size (Figure 3). Pathologic analysis of the specimen confirmed a hyalinized nodule with associated chronic inflammation, suggestive of a remote ectopic tubal pregnancy site.

The patient was seen for post-operative evaluation five weeks after the surgery and reported that her left-sided pelvic pain had vastly improved. She was cleared to resume sexual intercourse. She was seen again 12 weeks after the surgery for a well woman exam and reported complete resolution of her abdominal pain and dyspareunia.

## Discussion

This case demonstrates the workup of pelvic pain in a patient successfully treated with methotrexate for ectopic pregnancy. Despite a negative urine hCG, pathology demonstrated residual ectopic tissue within the fallopian tube. Given that the pain completely resolved after salpingectomy, we can deduce that the residual ectopic tissue was the cause of this patient’s chronic pelvic pain. A review of the literature yielded no data on the incidence of chronic pelvic pain following methotrexate treatment for ectopic pregnancy. Given that persistent pain is an indication for acute surgical intervention in the setting of suspected failed methotrexate treatment, it makes sense there is limited data on this outcome [5].

However, the patient’s presentation is similar to that of chronic ectopic pregnancy, a better-studied, yet still ill-defined, clinical outcome. There is no precise definition or universal criteria for chronic ectopic pregnancy, but most describe it as a process whereby trophoblastic tissue implants in the fallopian tube and causes chronic local inflammation and bleeding, frequently resulting in a pelvic mass. Chronic ectopic pregnancy similarly results in hCG levels that are low or undetectable [6]. For example, Drakopoulos et al. described a patient with the last menstrual period approximately eight weeks prior and a negative pregnancy test found to have hematosalpinx. Pathology confirmed a chronic tubal ectopic pregnancy as evident by chorionic villi [7]. However, our patient’s presentation differs significantly in that she had unresolved ectopic tissue months after successful treatment of the ectopic pregnancy with methotrexate. Kasaven et al. described a similar case of a patient successfully treated with methotrexate 12 months prior to workup for infertility. On diagnostic laparoscopy, she was found to have a 2 cm mass in the ampulla of the fallopian tube which pathology confirmed as a chronic ectopic pregnancy with scanty degenerated chorionic villi [8].

## Conclusions

Methotrexate treatment for ectopic pregnancy has well-known common adverse effects and potential complications. Providers should also consider unresolved ectopic tissue as a cause of chronic pelvic pain in patients treated with methotrexate. The case presented describes a patient who endured chronic pelvic pain for nearly seven months after medical management of ectopic pregnancy until definitive surgical management was performed. Further research needs to be done on the incidence of chronic pelvic pain following methotrexate treatment for ectopic pregnancy. The risk of chronic pelvic pain or chronic ectopic pregnancy may need to be considered during discussion with patients who choose medical management of ectopic pregnancy.

## References

[1] Lau S, Tulandi T (1999). Conservative medical and surgical management of interstitial ectopic pregnancy. Fertil Steril.

[2] Cecchino GN, Araujo Júnior E, Elito Júnior J (2014). Methotrexate for ectopic pregnancy: when and how. Arch Gynecol Obstet.

[3] Barnhart KT (2009). Clinical practice. Ectopic pregnancy. N Engl J Med.

[4] (2013). Medical treatment of ectopic pregnancy: a committee opinion. Fertil Steril.

[5] van Mello NM, Mol F, Adriaanse AH (2008). The METEX study: methotrexate versus expectant management in women with ectopic pregnancy: a randomised controlled trial. BMC Womens Health.

[6] O'Neill D, Pounds R, Vella J, Singh K, Yap J (2018). The diagnostic conundrum of chronic ectopic pregnancy: a case report. Case Rep Womens Health.

[7] Drakopoulos P, Pluchino N, Yaron M, Dällenbach P (2014). Chronic tubal ectopic pregnancy: a rare but challenging diagnosis. BMJ Case Rep.

[8] Kasaven LS, Shah A, Sadoon S (2019). Chronic tubal ectopic pregnancy following clinically successful methotrexate treatment for an acute ectopic: a review of the literature. J Obstet Gynaecol.

